# Effects of total sleep deprivation on performance in a manual spacecraft docking task

**DOI:** 10.1038/s41526-024-00361-z

**Published:** 2024-02-21

**Authors:** Sarah Piechowski, Lennard J. Kalkoffen, Sibylle Benderoth, Oliver T. Wolf, Jörn Rittweger, Daniel Aeschbach, Christian Mühl

**Affiliations:** 1 Institute of Aerospace Medicine, German Aerospace Center (DLR), Cologne, Germany; 2 Department of Cognitive Psychology, Faculty of Psychology, Ruhr University Bochum, Bochum, Germany; 3 Department of Pediatrics and Adolescent Medicine, University Hospital Cologne, Cologne, Germany; 4 Institute of Experimental Epileptology and Cognition Research, University of Bonn Medical Center, 53127 Bonn, Germany

**Keywords:** Human behaviour, Human behaviour

## Abstract

Sleep deprivation and circadian rhythm disruptions are highly prevalent in shift workers, and also among astronauts. Resulting sleepiness can reduce cognitive performance, lead to catastrophic occupational events, and jeopardize space missions. We investigated whether 24 hours of total sleep deprivation would affect performance not only in the *Psychomotor Vigilance Task* (PVT), but also in a complex operational task, i.e. simulated manual spacecraft docking. Sixty-two healthy participants completed the manual docking simulation *6df* and the PVT once after a night of total sleep deprivation and once after eight hours of scheduled sleep in a counterbalanced order. We assessed the impact of sleep deprivation on docking as well as PVT performance and investigated if sustained attention is an essential component of operational performance after sleep loss. The results showed that docking accuracy decreased significantly after sleep deprivation in comparison to the control condition, but only at difficult task levels. PVT performance deteriorated under sleep deprivation. Participants with larger impairments in PVT response speed after sleep deprivation also showed larger impairments in docking accuracy. In conclusion, sleep deprivation led to impaired *6df* performance, which was partly explained by impairments in sustained attention. Elevated motivation levels due to the novelty and attractiveness of the task may have helped participants to compensate for the effects of sleepiness at easier task levels. Continued testing of manual docking skills could be a useful tool both to detect sleep loss-related impairments and assess astronauts’ readiness for duty during long-duration missions.

## Introduction

Insufficient sleep duration and quality are common in many occupational contexts, especially when extended duties or shift work are required^1–3^. Sleep deprivation affects the performance of people working in health care^4^, transportation^5–8^, military, and also human space flight. Although the use of sleep-promoting drugs is common, astronauts sleep only about six hours per night, resulting in chronic sleep deprivation over time^9–11^. There are numerous reasons why sleep is particularly “lost in space”, including environmental (e.g., noise, altered light-dark cycle, hypoxia, and hypercapnia) and psychological (e.g., isolation, confinement, and stress) factors^12^. Moreover, mission demands can require irregular sleep-wake cycles of astronauts, such as slam-shifts, resulting in a high prevalence of circadian misalignment^13^. Sleepiness – in the occupational field often referred to as fatigue – is an important risk factor in work environments that require a continuously high level of performance to avoid potentially catastrophic outcomes^14^. In many operational contexts, sleepiness has been shown to impair performance and facilitate human error, increasing the risk of accidents^15–17^. Sleep-deprived pilots display degradations in psychomotor control, problem solving, and attention to flight instruments. Moreover, short involuntary lapses into sleep have been the cause of aviation accidents^18^.

In astronauts, a sleep duration of <6 h was correlated with impairments in sustained attention and a decrease in mood on the following day^11^. One example of sleep-related risks in space is the crash of the Progress space shuttle with Mir space station in 1997. The life-threatening accident occurred during a manual docking maneuver and fatigue was made out as one of the contributing factors^19^. Manual docking of a spacecraft is a mission-critical operational task in space and requires highly trained cognitive skills as well as motor skills in the control of an object with six degrees of freedom (DoF, Fig. 2). Because docking performance deteriorates fast without continuous training^20^, the *6df* simulation has been developed as a tool that facilitates skill assessment as well as autonomous training and maintenance for controlling six DoF^21,22^. Basner and colleagues have recently studied the relationship between cognitive performance in the *Cognition* test battery and operational performance in the *6df* docking task^23^. The strongest association was observed with performance in the Digit-Symbol Substitution Task (DSST), a measure of processing speed, visual tracking, and working memory. These domains are not only crucial to process instrument information during manual docking, but are also known to be sensitive to sleep loss^24,25^. Furthermore, there was a positive correlation between manual docking performance and sustained attention as measured by accuracy in the Psychomotor Vigilance Task (PVT). In sleep deprivation research, a decrease in sustained attention has been one of the most reliable effects^26^. Sleep-deprived individuals are consistently slower in responding to stimuli and more prone to errors of omission and commission^27^, therefore, the PVT is considered to be one of the most sensitive measures regarding sleep restriction^15^. Alertness is a critical factor for space mission safety^28^, as even small decrements in the ability to timely react to relevant stimuli can compromise task success.

Currently, it is still unclear whether or to what extent sleepiness-related decrements in experimental tests of cognitive and psychomotor performance relate to impairments in more complex operational tasks^29^. The higher demand of complex tasks might as well motivate individuals to apply additional effort to compensate for their sleepiness^30^. Strangman et al.^31^ for example detected compensatory cerebral responses to sleep deprivation, but no performance impairment in a simulated orbital docking task. Wong et al.^32^ attributed the lack of performance decrements during the course of 28 h of sleep deprivation to the novelty and motivational character of their grappling and docking task. However, there are few studies and only with a small number of participants that looked at the effects of sleep deprivation on spaceflight-relevant operational performance. Therefore, our study aimed at characterizing the influence of one night of total sleep deprivation (~24 h awake) on manual docking performance. Specifically, we hypothesized that docking accuracy as well as the progression through different levels of task difficulty will deteriorate after sleep deprivation in comparison to performance after normal sleep. Additionally, we assessed if impairment in sustained attention (as measured by the PVT) is a relevant factor to explain docking performance under sleep deprivation.

## Methods

### Participants

Sixty-six healthy individuals participated in our experiment. Two participants were excluded because they attended only one of the test sessions and two because of technical problems. The final sample consisted of 62 participants, 28 women and 34 men, aged between 18 and 39 (*M* = 24.84, SD = 4.69) years. Most participants were students recruited via university job web portals. Ahead of the study, participants completed a medical examination as well as questionnaires to rule out the presence of sleep problems (STOP-Bang questionnaire, Epworth Sleepiness Scale (ESS)), extreme personality traits (Freiburger Persönlichkeitsinventar (FPI)) and depression (Beck Depression Inventory (BDI)). Other exclusion criteria were pregnancy, smoking, drug use, relevant medication, and body mass index above 30 kg/m^2^.

### Study design

**Fig. 1:**
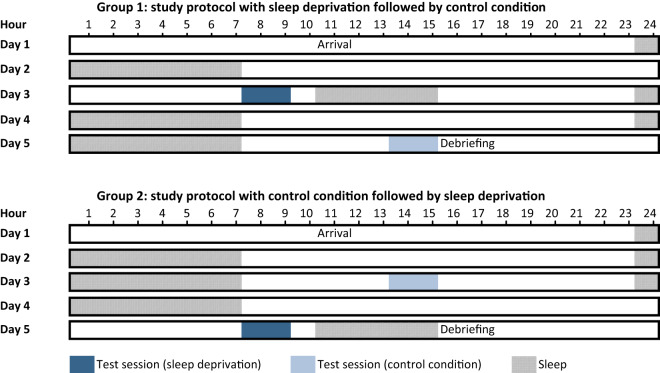
Sleep deprivation study protocol. All participants stayed in the laboratory for 5 days and completed two test sessions: one sleep deprivation condition and one control condition. The order of both conditions was counterbalanced and randomly assigned. The figure indicates the timing of scheduled sleep and test sessions for both groups.

The docking experiment was part of a larger laboratory study that was conducted in the Institute of Aerospace Medicine of the German Aerospace Center (DLR) in Cologne, Germany, from 2019 to 2020. The study followed a randomized counter-balanced cross-over design with a control condition and a sleep deprivation condition. At least one week before arrival at the Simulation Facility for Occupational Medicine Research (AMSAN), participants were required to follow a regular sleep protocol (23:00–07:00 h) in order to avoid sleep debt and circadian misalignment. Compliance was ensured via wrist actimetry (Philips Actiwatch Spectrum) and sleep diaries. Caffeine consumption was not allowed in the week before and during the study. Participants were randomly assigned to one of two groups with a counterbalanced order of experimental conditions (control, sleep deprivation). Participants spent five days and four nights in the laboratory in three-person teams. An illustration of the study protocol is provided in Fig. 1. Test sessions took place on days three and five. In the control condition, the test session was scheduled from 13:00 to 15:00 h and was preceded by an 8-h sleep episode (23:00–07:00 h). In the sleep deprivation condition the test session took place between 07:00 and 09:00 h following approximately 24 h of continuous, monitored wakefulness. Apart from the manual docking simulation task and the PVT reported here, test sessions consisted of a synthetic operational group task^33^ as well as a cognitive test battery, but these data will be presented elsewhere and were not within the scope of the current investigation. During the scheduled wake episodes illuminance was maintained at ~100 lux at the horizontal angle of gaze. The study was approved by the ethics committee of the medical association North-Rhine in Dusseldorf, Germany, and registered with the German register for clinical studies (www.drks.de) with the identifier DRKS00017789. All participants provided written informed consent prior to their participation.

### Docking simulation

**Fig. 2:**
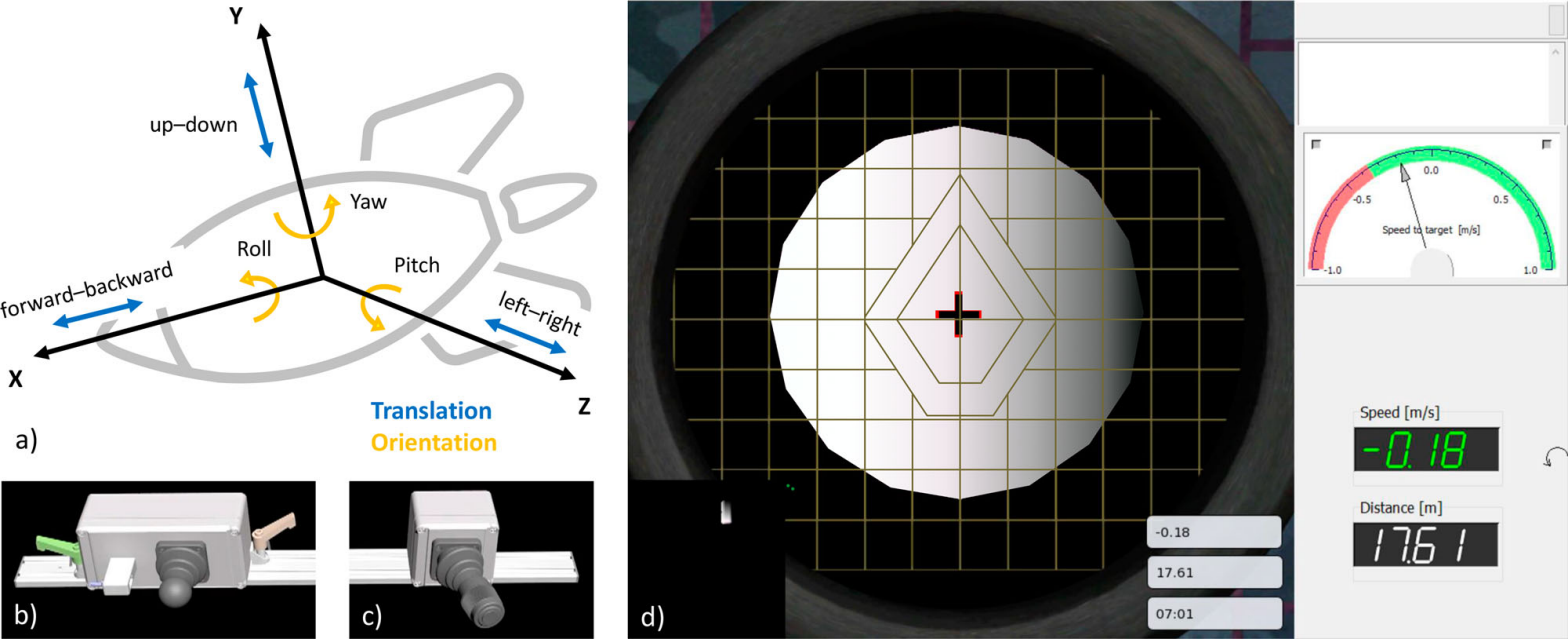
*6df* manual docking simulation. Spacecraft docking is based on the control of six degrees of freedom (**a**). Translation is controlled with the left hand control (joystick movement up-down and left-right, trigger movement forwards and backwards) (**b**), orientation with the right hand control (rotation of the joystick around its own axis for roll, movement of the joystick for pitch and yaw) (**c**). Screenshot of a level 4 *6df* docking trial showing the view from the spacecraft to the white space station (**d**). The black cross represents the target/docking port.

The *6df* tool is a computer-based simulation platform for the control of an object with six degrees of freedom (Fig. 2), in this case, the manual docking of a spacecraft to a space station^22^. Flight dynamics and controller responsiveness are based on the Russian docking training system TORU (Teleoperatiya Ruchnogo Upravleniya – teleoperated manual control) and the actual Soyuz spacecraft. However, the tool is not designed to be a realistic Soyuz simulation, but to teach the principles of control of any object in space in a generic fashion (Fig. 2). The *6df* software was developed by SpaceBit GmbH (Eberswalde, Germany) and hand controls were produced by Koralewski Industrie-Elektronik oHG (Hambühren, Germany). The left hand control operates the translational degrees of freedom and the right hand control operates all rotational degrees of freedom. In this experiment, we used a shortened adaptive version of *6df*. It consisted of eleven docking task designs from five difficulty levels. For level 1, only one degree of freedom had to be controlled (e.g. “move the spacecraft to the right”), for level 2, it was two degrees of freedom (e.g. “move the spacecraft right and up simultaneously”). Level 3 required to orientate and stabilize the spacecraft in a safety distance from the station; both hand controls were necessary. Level 4 also included this stabilization phase, but continued with an approach to the station and docking contact. Level 5 represented a standard docking maneuver including a curved flight-around, stabilization, approach, and docking contact.

Participants had no previous experience or training with the docking simulation. Prior to the first test session (either sleep deprivation or control condition), they received written instructions with general information regarding the *6df* tool, task design, and performance feedback. Each of the two test sessions started with a short instructional film on the use of the hand controls. Additionally, before each docking trial, an illustrated text with specific instructions was presented. After completion of a trial, performance feedback following TORU methodology was given^34^. This included single parameters such as forward speed, pitch, bank, and yaw, as well as an aggregated general performance measure (accuracy), with zero being the worst and one the best possible accuracy. Every session started with the same trial on level 4 out of 5; a docking maneuver excluding the curved flight-around to reach the stabilization point. After this first trial, the difficulty level of the next trial was always chosen based on the participant’s performance: a docking maneuver was deemed successful if the accuracy was at least .85, and of a good quality if at least .95. Therefore, if the accuracy score was below .85, an easier trial was presented next. For accuracy scores between .85 and .95, the trial was repeated and for scores ≥.95, a more difficult trial was presented. Each single docking trial lasted up to ten minutes without instructions and feedback, depending on level and participant’s speed. After 35 minutes, the session was terminated following completion of the current trial. Therefore, number and level of completed docking trials varied between participants. By this procedure, every participant was presented with appropriate task difficulties according to their skill level, even without previous training in the task.

### Psychomotor Vigilance Task

Participants performed a 10-minute version of the PVT^35,36^. The task was to react to the appearance of a millisecond counter as fast as possible by pressing a button with the thumb of the dominant hand (handedness, percent of participants: 6.45% left, 93.55% right). Upon response, the counter stopped and presented the reaction time as feedback for 1 second. Without a response, the counter timed out after 10 seconds. The interstimulus interval varied pseudo-randomly between 2 and 10 seconds. Only responses greater than 130 milliseconds were considered valid and lapses were defined as a response time exceeding 500 milliseconds. The PVT was implemented in Matlab using the Psychophysical toolbox^37^ on a Windows desktop machine. Responses were recorded via an Arduino-based response box^38^. The setup was calibrated, showing that logged reaction times deviated from externally recorded stimulus-initiated “button-presses” (using a photo sensor to response box loop) with an acceptable standard deviation smaller 1 millisecond and a bias of 9 milliseconds, mainly related to stimulus buildup time as measured at screen center^39^. Due to its high sensitivity to sleep loss^26^, the averaged response speed per session, i.e., reciprocal response time (RT^−1^), and the number of lapses were chosen as outcomes.

### Statistical Analysis

IBM SPSS Statistics 26 was used for raw data preprocessing and R 4.0.2/R Studio for data analysis. All tests were carried out two-sided and with a significance level of *α* = 0.05. When applicable, normal distribution of variables or residuals was verified via visual inspection of Q-Q plots and histograms. A total number of 856 docking trials were absolved. We excluded 56 trials that were discontinued because the participant maneuvered out of the station’s reach (distance > 200 meters), resulting in a final sample of 800 valid docking trials.

For mixed models, conditional and marginal pseudo-*R*^*2*^ were computed with the MuMIn package for R^40^ as an effect size estimate proposed by Nakagawa et al.^41^. Conditional *R*^2^ (*R*^2^_c_) is interpreted as the variance explained by the entire model including random effects, whereas marginal *R*^2^ (*R*^2^_m_) represents the variance explained by fixed effects only. For nonparametric analyses, $r=Z\div\sqrt{N}$ was computed as effect size^42^.

To check if the sleep deprivation manipulation actually induced sleepiness, we compared self-reported subjective sleepiness between conditions using a linear mixed model. For this purpose, participants completed the Karolinska Sleepiness Scale (KSS)^43,44^ at the beginning of each test session. The KSS is a single-item questionnaire that indicates situational sleepiness on a 9-point Likert scale, ranging from 1 (extremely alert) to 9 (extremely sleepy). The effect of sleep deprivation on PVT response speed and number of lapses (following log transformation) was also analyzed using linear mixed models.

To test the effect of sleep deprivation on docking accuracy, we computed a linear mixed model with accuracy as the dependent variable and participants as random intercept. As fixed effects, we included *6df* level of difficulty, session (first or second), condition (control or sleep deprivation) as well as age and gender. For docking accuracy scores an inverse log transformation was used to achieve normal distribution of residuals. Levels 1 and 2 as well as 4 and 5 were merged for this analysis because of similar task demands and the small number of observations in levels 1 and 5. In a second step, we added the susceptibility to sleep deprivation (as measured by PVT response speed or number of lapses) and the interaction of susceptibility with condition to the model. Susceptibility scores were obtained by subtracting control condition (CC) PVT performance from sleep deprivation (SDC) PVT performance. Larger impairments in PVT performance due to sleep loss were interpreted as higher susceptibility to sleep loss. Worse performance after sleep deprivation relative to the control condition is indicated by negative SDC-CC difference values in the case of response speed, and positive SDC-CC difference values in the case of lapses.

Next to the accuracy of the docking maneuvers, we also considered participants’ progress through the levels of the adaptive *6df* program during each session. For this purpose, we looked at the level of the highest successfully completed trial (accuracy ≥ .85) in each session. The highest achieved level could vary between 1 (if the only trial a participant was able to solve with sufficient accuracy was on level 1) and 5 (if a participant advanced from level 4 and achieved sufficient accuracy also in a level 5 trial). Because the data was not normally distributed, we chose the Wilcoxon signed-rank test to compare the highest levels between control and sleep deprivation condition as well as between first and second session.

### Reporting summary

Further information on research design is available in the Nature Research Reporting Summary linked to this article.

## Results

### Sleep deprivation decreased sustained attention

After sleep deprivation, participants reported to feel significantly sleepier (KSS: *M* = 7.52, SD = 1.54) than in the control condition (*M* = 3.53, SD = 1.61; *F*(1, 61) = 223.15, *p* < 0.001, *R*^2^_m_ = 0.62, *R*^2^_c_ = 0.66). Additionally, sleep deprivation compromised PVT performance: response speed deteriorated significantly (*F*(1, 61) = 103.85, *p* < 0.001, *R*^2^_m_ = 0.19, *R*^2^_c_ = 0.78) after sleep deprivation (*M* = 3.36 s^−1^, SD = 0.65) in comparison to control condition performance (*M* = 3.89 s^−1^, SD = 0.45). Also, the number of lapses was significantly larger (*F*(1, 61) = 95.93, *p* < 0.001, *R*^2^_m_ = 0.30, *R*^2^_c_ = 0.61) after sleep deprivation (*M* = 9.40, SD = 9.76) than after normal sleep (*M* = 1.76, SD = 4.02).

### Sleep deprivation impaired accuracy in a manual docking task

**Fig. 3:**
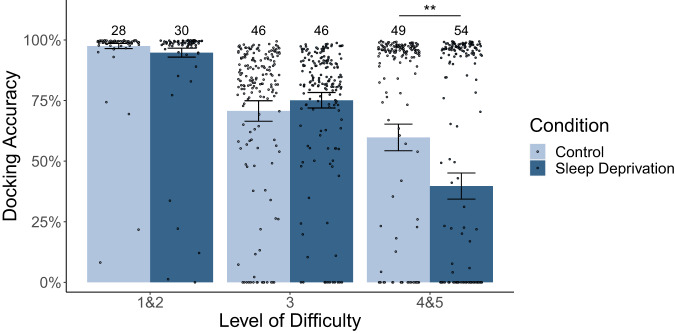
Average docking accuracy scores under control and sleep deprivation conditions for each level of difficulty (each session started on level 4). Error bars represent the standard error, the number of participants in each category is indicated above the bars. Dots show individual data points. Trials that were discontinued because the participant maneuvered out of the station’s reach (distance >200 m) were excluded.

Participants completed on average 6.32 docking trials with an average duration of 4.38 minutes in the control condition and 6.58 trials of 4.26 minutes duration in the sleep-deprived condition. The average accuracy score over all trials was *M* = 0.76 (SD = 0.33). Figure 3 depicts docking accuracy in both conditions for each difficulty level. The linear mixed model (detailed model results are given in Supplementary Table 1) revealed significant main effects of level (*F*(2, 776.68) = 349.15, *p* < 0.001) and session (*F*(1, 746.50) = 81.62, *p* < 0.001), as well as an interaction between level and session (*F*(2, 736.32) = 4.97, *p* = 0.007). Participants improved their docking accuracy from the first (*M* = 0.73, SD = 0.35) to the second session (*M* = 0.79, SD = 0.32) and docking accuracy decreased with increasing difficulty level. There was no significant interaction of condition with session (*F*(1, 52.99) = 2.60, *p* = 0.11), indicating that effects of learning across consecutive sessions did not skew the results attributed to condition. Importantly, there was a significant main effect of condition (*F*(1, 747.76) = 9.15, *p* = 0.003). This result is consistent with our hypothesis that accuracy decreases after sleep deprivation (*M* = 0.74, SD = 0.35) in comparison to control condition performance (*M* = 0.78, SD = 0.32). Whereas there was no trifold interaction (*F*(2, 781.14) = 0.97, *p* = 0.38), we observed a significant interaction of condition and level (*F*(2, 736.17) = 3.94, *p* = 0.02). Docking accuracy was significantly higher in men (*M* = 0.79, SD = 0.13) than in women (*M* = 0.74, SD = 0.08; *F*(1, 49.37) = 11.97, *p* = 0.001) and decreased with increasing participant age (*F*(1, 49.65) = 8.95, *p* = 0.004). The proportion of explained variance can be described by *R*^2^_c_ = 0.65 for the whole model and *R*^2^_m_ = 0.46 for fixed effects only.

To further delineate the origin of the interaction between condition and level (see also Fig. 3), post-hoc contrasts between sleep deprivation and control condition for each level were performed by using the emmeans package for R^45^. *P*-values were adjusted for multiple testing according to the false discovery rate method by Benjamini and Hochberg^46^. There was no significant effect of condition on docking accuracy on levels 1 and 2 (*t*(739) = −1.79, *p* = 0.11) or level 3 (*t*(764) = 0.12, *p* = 0.91). Instead, accuracy in levels 4 and 5 decreased after sleep deprivation (*t*(745) = −3.63, *p* < 0.001).

### Relationship with impairments in sustained attention

When adding the susceptibility to sleep loss in terms of PVT response speed (SDC-CC difference) to the model, the significant main effect of condition vanished (*F*(1, 729.04) = 0.06, *p* = 0.81). However, the effect of condition on docking accuracy remained dependent on the difficulty level (*F*(2, 735.32) = 3.52, *p* = 0.03). Additionally, there was a significant interaction between condition and susceptibility (*F*(1, 726.00) = 6.33, *p* = 0.01). Participants with higher susceptibility to sleep deprivation (large increase in PVT response time in the sleep deprivation condition compared with the control condition) also showed a larger difference of docking accuracy between conditions. The proportion of explained variance for this extended model was *R*^2^_c_ = 0.66 and *R*^2^_m_ = 0.46 for fixed effects only. When including susceptibility in terms of the number of lapses during the PVT, the main effect of condition on docking accuracy disappeared likewise (*F*(1, 732.41) = 1.47, *p* = 0.23). The interaction of condition and level persisted (*F*(2, 734.83) = 3.60, *p* = 0.03), but there was no significant interaction between condition and susceptibility to sleep loss (*F*(1, 726.53) = 3.43, *p* = 0.06). The proportion of explained variance for this second extended model was *R*^2^_c_ = 0.65 and *R*^2^_m_ = 0.46 for fixed effects only.

### Level progression was not impaired after sleep deprivation

**Fig. 4:**
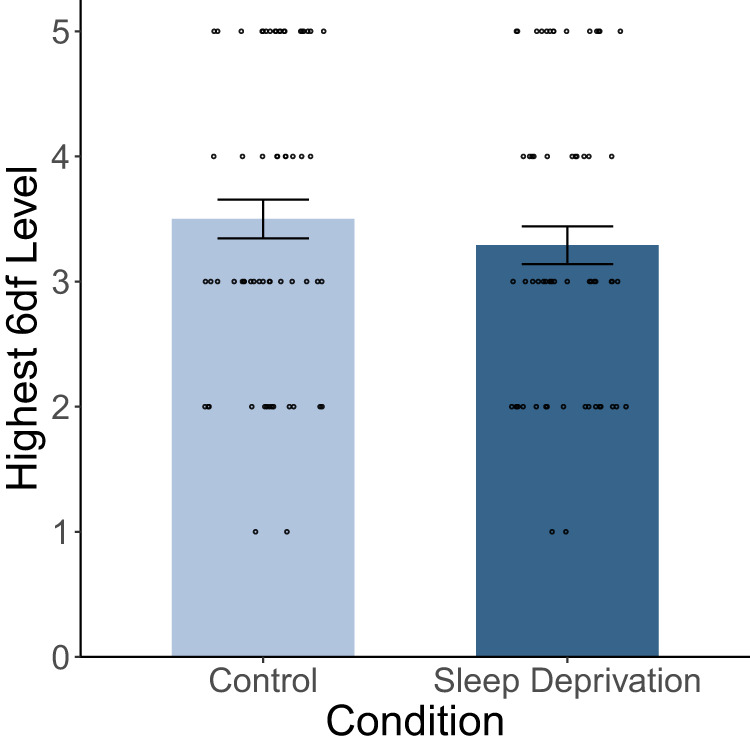
Average highest successfully completed *6df* level per condition. Dots depict individual data points; error bars indicate the standard error.

Of all 800 trials that were analyzed, 3.25% were of level 1 difficulty, 14.25% level 2, 43.88% level 3, 28.75% level 4, and 9.87% level 5. Looking at the progress through the *6df* program instead of accuracy (Fig. 4), participants’ performance also improved significantly from the first to the second session (*V* = 13.50, *p* < 0.001, *r* = 0.76). The average highest successfully completed level increased from *M* = 2.95 (SD = 1.06, Median = 3) to *M* = 3.84 (SD = 1.19, Median = 4). Participants on average reached a slightly lower level after sleep deprivation (*M* = 3.29, SD = 1.19, Median = 3) than in the control condition (*M* = 3.50, SD = 1.22, Median = 3). However, this difference was not statistically significant (*V* = 531.50, *p* = 0.18).

## Discussion

Astronauts in space usually sleep less^9,10^ than the seven hours that – according to consensus reports – are needed to maintain health and cognitive performance in adults^47^. Whereas sleep loss is known to impair performance in a multitude of cognitive tests, the goal of the present study was to investigate the potential effect of sleep deprivation on simulated manual spacecraft docking – a complex and mission-critical operational task – as well as to identify the relationship with performance in the PVT as a standard measure sensitive to sleep loss.

Our results revealed a detrimental effect of sleep deprivation on accuracy in the complex manual docking simulation. Although participants were able to compensate for their sleepiness in easier docking trials (levels 1–3), they were significantly impaired during the more difficult trials at levels 4 and 5. Whereas levels 1–3 include only single components of manual control, the difficult trials represent a full docking maneuver as it is required in an operational context. This observation stands in contrast to previous studies that were not able to detect impairments in similarly complex tasks after sleep deprivation, possibly due to small sample sizes^31,32^ or the lack of a control condition without sleep deprivation^32^. In contrast to docking accuracy, we found no significant difference between conditions regarding the highest level participants reached. This might be explained by the limited variance of levels offered in the simulation program. Participants’ age and gender had a significant influence on docking accuracy. However, it should be noted that the age range in this study was very limited – most participants were 20 to 30 years old. In previous studies, similar age effects were demonstrated for learning speed with the *6df* learning program, whereas the results were mixed in regard to gender^21,48^. Gender differences may have arisen from potential disparities in gaming experience of participants, as motor practice with video game joysticks might facilitate the familiarization with the hand controls used for the *6df* simulation.

As expected, participants showed a substantial decline in PVT response speed and an increase in the number of lapses when sleep-deprived. Measures of sustained attention are generally considered to be most sensitive to sleep loss^24,27^. Novelty and attractiveness of the docking simulation are likely reasons for the absence of larger performance decrements across difficulty levels^32^. Heightened motivation might have helped participants to compensate for their sleepiness at least partly. High-level complex skills are generally assumed to be less affected by sleep deprivation compared to monotonous, less demanding tasks, because they enhance motivation and effort^30^. The PVT however is a very monotonous task^49^. Additionally, speed measures seem to be more susceptible to total sleep deprivation than accuracy measures^24,50^. If individuals are given sufficient time to complete a motivating task, the slowing of cognitive functioning due to sleep loss can be compensated to a large degree^30^. Participants in the 3D sensorimotor navigation study by Strangman et al.^31^ displayed no performance decrements, but reported the docking to be more effortful under sleep deprivation. Accordingly, functional magnetic resonance imaging revealed compensatory cerebral responses to sleep deprivation in cortical regions associated with visuospatial processing, memory, and attention.

The degree of performance impairment in response to sleep loss is subject to substantial inter-individual differences that are stable and trait-like^36,51–54^. Therefore, the PVT has been used to classify individuals regarding their vulnerability or susceptibility to sleep loss^55,56^. Performance deficits after sleep deprivation are oftentimes attributed to impairments in sustained attention. The latter is seen as prerequisite of more complex cognitive processes^27,30^. In our study, the inclusion of the impairment in sustained attention due to sleep deprivation (measured as the SDC-CC difference in response speed and number of lapses) dissolved the main effect of sleep deprivation on docking accuracy. However, a significant interaction of level and sleep deprivation persisted. For response speed (but not number of lapses), the interaction with condition indicated that participants who reacted to sleep deprivation with larger performance decrements in PVT response speed also displayed larger performance decrements in docking accuracy. Whereas sustained attention is indeed an important component of complex operational performance under sleep loss, it can only partly account for impairments in the more complex docking task. The PVT has already been proposed as a short test to evaluate fitness for duty prior to an operational task. For example, the PVT predicted performance decline in a luggage screening task that is based heavily on sustained attention^57^, but was not indicative of performance impairment in a driving simulation^58^. On the ISS, the PVT is part of the standard measures used to monitor astronauts’ cognitive functioning during their missions. Although the PVT is not sufficient to predict operational performance, it may be useful as a first indicator to timely detect possible performance decrements due to fatigue. However, our results also underline the need for operational task designs to assess performance or readiness for duty in safety-critical contexts.

This study has several limitations. One of them is the special adaptive task design. Because number and difficulty of completed tasks varied, performance was not easily comparable between participants. However, this version of *6df* allowed for the investigation of a complex task in completely untrained novices, while being reasonably challenging for every initial skill level. However, the lack of previous training with the docking simulation can also be seen as a limitation. The resulting considerable performance variance between participants might have partially masked the effects of sleep deprivation. Sleep deprivation itself leads to increased variability within individuals due to state instability as well as between individuals due to differences in vulnerability to sleep loss, which poses the risk of missing performance decrements if sample size is limited^25^. Although novice students differ from an astronaut population (e.g. professionalized training), they allowed for a larger sample size and a controlled experimental design. In our study, we observed an order effect which likely resulted from the benefits of continued training across the two test sessions. The use of a counterbalanced cross-over design protected against some of these influences masking the sleep deprivation effect. Moreover, our analysis did not reveal a significant interaction between test session and condition, indicating learning occurred even under sleep deprivation. Previous studies also evidenced continued learning in a similar task during sleep deprivation^32^. However, it is still unclear if and to what extent learning may be attenuated under conditions of sleep loss. Our study and task design were not suitable to quantify learning under sleep loss. In this context, it will also be interesting to investigate the role of training and its potential interaction with the effects of sleep loss in future studies of docking performance. Whereas training diminishes the novelty of the task, exhaustive training is expected to be protective against performance errors^29^. Furthermore, the operational importance of a manual docking maneuver in professionals should have a high impact on motivation, counteracting the decrease in arousal due to sleepiness. Nevertheless, for a highly safety-relevant task like manual docking, even small performance decrements can have serious consequences. In the aviation domain, less than six hours of sleep already pose a substantial risk factor^17^, therefore, further investigation of performance in complex operational tasks is necessary to achieve a comprehensive risk assessment. It should be noted that sleep deprivation and control measurements took place at different times of the day, therefore, it is possible that differences in circadian phase may have contributed to the observed results. However, a 6-h phase difference during the early part of the biological day (defined as the phase range of melatonin’s absence from the blood) is expected to have a rather small effect on measures of cognitive performance – including attention – as revealed in forced desynchrony studies^59,60^. Our results might not generalize to chronic sleep deprivation, which is highly prevalent in space missions^9,11^ and many other occupational contexts^61^. The effects of recurrent partial sleep deprivation on cognitive performance might be even more pronounced compared to total sleep deprivation^62^ and it is unclear to what extent motivational factors may be protective under these circumstances. Performance on long-term missions to moon and Mars will likely be more vulnerable, because fewer resources are available to buffer the effects of sleepiness and circadian misalignment^29^. Adaptive training systems such as *6df* will be important tools during future long-term missions to help preserve astronauts’ cognitive and operational functioning in a motivating and autonomous manner.

The aim of this counterbalanced cross-over study was to assess the influence of ~24 h total sleep deprivation on performance in a mission-relevant operational task and a sustained attention task. Our results demonstrate performance decrements in a complex manual docking simulation that were explained partly by decrements in sustained attention. Docking performance was impaired in difficult trials, but not in easier ones – possibly because the task’s novelty and engaging quality was enough to overcome impairments during lower task demand. However, even small decrements in accuracy could have catastrophic consequences in safety-critical tasks, especially when various stressors accumulate. Future studies should assess the influence of exhaustive training on the susceptibility to sleep loss. Operational performance measures like those gathered from the *6df* task could be helpful tools to assess readiness for duty under sleep deprivation during long-duration missions. The susceptibility to sleep deprivation as measured by the PVT is useful for the prediction and early detection of performance decrements in such more complex tasks.

### Supplementary information

Supplementary Table 1

Reporting Summary

## Data Availability

The data that support the findings of this study are available in the Open Science Framework repository at https://osf.io/e5xrg/.

## Code availability

The analysis code to reproduce the results of this study is available in the Open Science Framework repository at https://osf.io/e5xrg/.
