# Supplementary material for: Effects of total sleep deprivation on performance in a manual spacecraft docking task
Source: NPJ Microgravity. 2024 Feb 21;10:21. doi: 10.1038/s41526-024-00361-z (PMC10881462; doi:10.1038/s41526-024-00361-z)
Supplement: Supplementary file 1 — Supplementary Table 1 [file 41526_2024_361_MOESM1_ESM.pdf]

Supplementary Table 1. Detailed linear mixed effects model results for *6df* performance

	β	*95% CI*	*t*	*p*
**Basic model**				
Log 6df Accuracy[1]				
Intercept	-1.28	-1.88, -0.69	-4.23	< .001
Sleep Deprivation	0.31	0.02, 0.59	2.12	.04
Level 3	1.40	1.21, 1.59	14.34	< .001
Level 4&5	1.60	1.37, 1.83	13.63	< .001
Session 2	-0.23	-0.55, 0.10	-1.37	.17
age	0.03	0.01, 0.05	2.99	< .01
gender	0.36	0.15, 0.56	3.46	< .001
Sleep Deprivation * Level 3	-0.20	-0.46, 0.06	-1.53	.13
Sleep Deprivation * Level 4&5	0.16	-0.15, 0.47	1.00	.32
Sleep Deprivation * Session 2	-0.29	-0.83, 0.26	-1.03	.30
Level 3 * Session 2	0.09	-0.21, 0.40	0.62	.54
Level 4&5 * Session 2	-0.04	-0.38, 0.30	-0.23	.82
Sleep Deprivation * Level 3 * Session 2	0.06	-0.38, 0.50	0.27	.79
Sleep Deprivation * Level 4&5 * Session 2	-0.20	-0.72, 0.31	-0.77	.44
**Extended model including susceptibility to sleep deprivation[2] (based on response speed)**				
Log 6df Accuracy				
Intercept	-1.27	-1.91, -0.64	-3.95	< .001
Sleep Deprivation	0.19	-0.11, 0.49	1.27	.21
Level 3	1.41	1.22, 1.60	14.45	< .001
Level 4&5	1.61	1.38, 1.84	13.77	< .001
Session 2	-0.22	-0.55, 0.11	-1.32	.19
Susceptibility (Speed)	0.08	-0.18, 0.33	0.58	.56
age	0.03	0.01, 0.06	2.99	< .01
gender	0.36	0.16, 0.57	3.47	< .001
Sleep Deprivation * Level 3	-0.20	-0.45, 0.06	-1.52	.13
Sleep Deprivation * Level 4&5	0.15	-0.16, 0.46	0.95	.34
Sleep Deprivation * Session 2	-0.27	-0.82, 0.28	-0.97	.33
Level 3 * Session 2	0.09	-0.21, 0.39	0.61	.55
Level 4&5 * Session 2	-0.04	-0.37, 0.30	-0.22	.83
Sleep Deprivation * Susceptibility (Speed)	-0.22	-0.39, -0.05	-2.51	.01
Sleep Deprivation * Level 3 * Session 2	0.05	-0.38, 0.49	0.24	.81
Sleep Deprivation * Level 4&5 * Session 2	-0.22	-0.73, 0.29	-0.84	.40
**Extended model including susceptibility to sleep deprivation (based on number of lapses)**				
Log 6df Accuracy				

Intercept	-1.27	-1.88, -0.67	-4.12	< .001
Sleep Deprivation	0.24	-0.06, 0.53	1.56	.12
Level 3	1.40	1.21, 1.60	14.38	< .001
Level 4&5	1.60	1.37, 1.83	13.66	< .001
Session 2	-0.22	-0.55, 0.11	-1.31	.19
Susceptibility (Lapses)	0.002	-0.02, 0.01	-0.39	.70
age	0.03	0.01, 0.05	2.98	< .01
gender	0.36	0.16, 0.56	3.46	< .001
Sleep Deprivation * Level 3	-0.19	-0.45, 0.06	-1.49	.14
Sleep Deprivation * Level 4&5	0.16	-0.15, 0.47	1.01	.32
Sleep Deprivation * Session 2	-0.27	-0.82, 0.29	-0.94	.35
Level 3 * Session 2	0.09	-0.21, 0.39	0.59	.55
Level 4&5 * Session 2	-0.04	-0.38, 0.30	-0.24	.81
Sleep Deprivation * Susceptibility (Lapses)	0.008	-0.0004, -0.02	1.85	.06
Sleep Deprivation * Level 3 * Session 2	0.06	-0.38, 0.49	0.25	.80
Sleep Deprivation * Level 4&5 * Session 2	-0.22	-0.73, 0.30	-0.83	.41

Notes. [1]Due to log-transformation, higher transformed accuracy values indicate lower performance. [2]Susceptibility scores were computed by subtracting control condition PVT performance from sleep deprivation PVT performance (as PVT outcomes reciprocal response speed and number of lapses were used respectively).
